# Conclusions from a behavioral aging study on male and female F2 hybrid mice on age-related behavior, buoyancy in water-based tests, and an ethical method to assess lifespan

**DOI:** 10.18632/aging.102242

**Published:** 2019-09-11

**Authors:** Julia Adelöf, Jaime M. Ross, Stanley E. Lazic, Madeleine Zetterberg, John Wiseman, Malin Hernebring

**Affiliations:** 1 Department of Clinical Neuroscience, Institute of Neuroscience and Physiology, Sahlgrenska Academy at the University of Gothenburg, Gothenburg 41390, Sweden; 2 Discovery Biology, Discovery Sciences, R&D AstraZeneca, Gothenburg, Mölndal 43153, Sweden; 3 Department of Genetics, Paul F. Glenn Center for the Biology of Aging, Harvard Medical School, Boston, MA 02215, USA; 4 Department of Neuroscience, Biomedicum, Karolinska Institutet, Stockholm 17165, Sweden; 5 Quantitative Biology, Discovery Sciences, R&D AstraZeneca, Cambridge CB4 0WG, UK; 6 Current address: Prioris.ai Inc., Ottawa K2P 2N2, Canada

**Keywords:** F2 hybrid mice, aging, sex comparison, exploratory activity, water-based behavioral tests

## Abstract

Due to strain-specific behavioral idiosyncrasies, inbred mouse strains are suboptimal research models for behavioral aging studies. The aim of this study is to determine age-related behavioral changes of F2 hybrid C57BL/6NxBALB/c male and female mice. Lifespan was followed (n_males_=48, n_females_=51) and cohorts of mature adult (7 months), middle-aged (15 months), and old mice (22 months of age; n=7-12 per group) were assessed regarding open-field activity, exploration, passive avoidance learning/memory, and depressive-like behavior. We found that both males and females demonstrated decreased exploratory behavior with age, while memory and depressive-like behavior were maintained. Females exhibited enhanced depressive-like behavior compared to males; however, a correlation between fat mass and swimming activity in the test directly accounted for 30-46% of this behavioral sex difference. In addition, we suggest a method to qualitatively estimate natural lifespan from survival analyses in which animals with signs of pain or severe disease are euthanized. This is, to our knowledge, the first behavioral study to consider both sex and aging in hybrid mice. We here define decreased exploratory behavior as a conserved hallmark of aging independent of sex, highlight the effect of buoyancy in water tests, and provide a method to assay lifespan with reduced animal suffering.

## INTRODUCTION

Mice are the leading mammalian model system for studying genetic effects on cognitive function and are well-suited model organisms for gerontological research with their relatively short lifespan and economic husbandry. Traditionally, mouse studies are conducted with inbred strains with the advantage of having less variation by genetic homogeneity and stable characteristics [1]. However, inbred strains can be problematic for aging research since they develop strain-specific maladies with advancing age and thus studies that intend to determine anti-aging effects might only pick up processes specifically targeting these conditions. Inbred strains also demonstrate age-related behavioral idiosyncrasies, *e.g*. coordination, learning capacity, and anxiety-like behavior, which can limit findings to the specific strain used [2, 3]. To ensure that these factors do not affect the results, it is considered favorable to use hybrid mice [4–6]. F2 hybrids are genetically similar but never uniformly homozygous, which reflects heterogenetic populations better than inbred mice and improves the extrapolation from mice to humans [5, 7].

There is a male sex bias in all biomedical disciplines [8, 9], although several studies confirm a sex difference in behavioral testing of mice [10–12]. Male rodents are dominant models of pharmaceutical discovery and testing, including several anxiolytic and anti-depressive drugs, despite the majority of recipients being women [13, 14]. Additionally, there are known sex differences in uptake and effect of psychotropic drugs, as well as symptoms and adverse side effects in humans [15–17]. Female exclusion has been rationalized by menstrual fluctuations interfering with behavioral data; however, a number of studies have shown that while the estrous cycle increases variability within female cohorts, behavioral differences between the sexes are independent of estrous cycle effects [18, 19].

Behavioral studies consisting of several complimentary tests allow for stronger phenotypic interpretations [20–22]. General behavior, locomotor activity, and exploratory behavior are easily assessed by open-field testing. Activity in the open-field and especially exploratory behavior of both male and female C57BL/6J mice [23–26] has been shown to decline with age and can fundamentally influence other behavioral testing during the aging process [23].

A substantial body of literature reports an aging effect in learning and memory, predominately assessed by spatial tests like Morris water, radial, and Barnes mazes [as reviewed in 27–29]. Decreased performance in these tests has been reported to correlate with female estrous cycle decay and aging [10, 26, 30, 31]. Additionally, sex differences in learning and memory have been found with spatial, cued, and water-based cognition tests, in which females performed poorer than males [30, 32–35]. Spatial reference tests, however, are unsuitable for sex comparisons in rodents since females and males are known to use different cues for navigation, and thus different parameters of these spatial tasks often favor either sex [36–39]. Interestingly, females and males perform equally well in non-spatial learning and memory tests, such as object memory consolidation [23, 30] and active avoidance tests [6, 10, 35].

The forced swim test is one of the most commonly used tests to assess depressive-like behavior by recording the activity of mice placed in water tanks. Immobility is considered a measurement of despair and is frequently used for anti-depressant screening in mice [40–43]. In addition to age [26], several factors such as strain, sex, and handling of mice can influence forced swim test performance [44, 45].

In this work, we analyzed the behavior of male and female C57BL/6N×BALB/c F2 hybrid mice as they age, by following the lifespan of littermates (n_males_=48, n_females_=51) and subjecting cohorts of mature adult (7 months), middle-aged (15 months), and old mice (22 months of age; n=7-12 per group) to behavioral phenotyping. To our knowledge, this is the first behavioral study to consider both sex and aging in mice with a hybrid background.

## RESULTS

### An estimation of C57BL/6N×BALB/c F2 natural lifespan

C57BL/6N×BALB/c F2 female and male hybrid mice were followed in a lifespan study, strictly following Swedish animal ethics regulations; if an animal displayed signs of pain or severe disease the animal was euthanized. The fate of the animals in the study is displayed in Table 1. The three shortest-lived and longest-lived animals are included as an indication of onset of death and maximum lifespan. As depicted, 15% of the males and 25% of the females were found dead in the cage without previous signs of pain or severe disease and thus died from intrinsic causes, generally defined as natural deaths in survival analyses. No autopsies were made but brief descriptions of extraordinary physical features were noted (e.g. enlarged spleen and various tumors; Supplementary Table 1).

**Table 1:** C57BL/6N×BALB/c F2 lifespan experiment.

	**Number of animals**	**Part of cohorts**	**Euthanized**	**Natural deaths**	**% Natural death**	Onset of death*	**3 most long lived**
♂Survival	48	28	13	7	15	311/312/613	860**/911**/919
♀Survival	51	29	9	13	25	352/406/560	966/1001/1051

*3 first animals to die from natural causes

**Removed due to pain or severe disease

**Figure 1:**
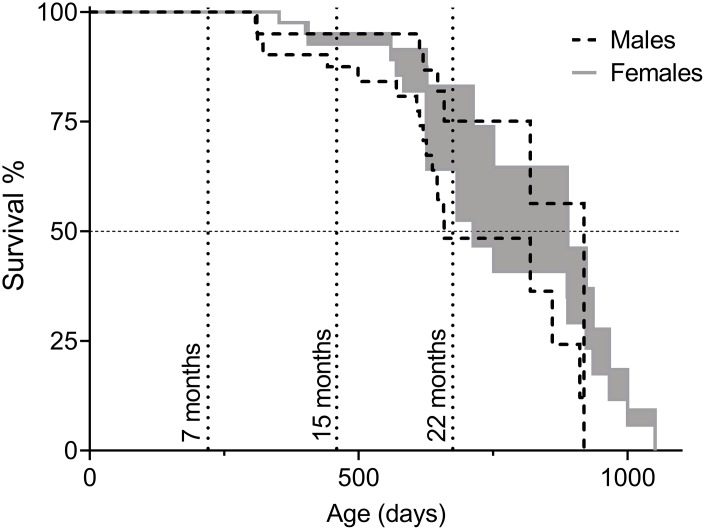
**Survival of C57BL/6N×BALB/c F2 hybrid male and female mice presented as intervals of natural lifespan.** The lower limit of the lifespans, referred to as “Survival Curve A” in Table 2, was obtained by considering euthanization of animals as the same fate as natural death and the upper limit of the lifespans, referred to as “Survival Curve B” in Table 2, by considering euthanized animals as healthy upon removal (censored) and only animals that died of intrinsic causes were counted as deaths. Animals in the cohorts for behavioral assessment were included in the survival analysis until the time of their first test (7, 15, or 22 months of age). Timepoints are indicated by dotted vertical lines. Total number of animals: n_males_=48, n_females_=51.

For lifespan analysis, two survival curves per sex were made. In Survival Curve A, euthanization was counted as natural death, while in Survival Curve B euthanized animals were considered healthy when removed from the study. Since animals of severe disease are likely to live longer than the onset of disease, but not as long as healthy littermates, Survival Curve A is an underestimation of natural lifespan and Survival Curve B is an overestimation. Taking both curves into account provides an interval of natural lifespan (Figure 1). Data from Survival Curves A and B are presented in Table 2. There were no statistically significant differences when comparing lifespan of male and female F2 hybrids, though trends of female longevity over male were observed on mean and median lifespan, as well as on the age of the three longest-lived animals (Table 1).

**Table 2:** Lifespan analysis C57BL/6N×BALB/c F2 to generate interval of natural lifespan in days.

	Mean lifespan*	**50% survival**	7 months’ survival**	15 months’ survival**	22 months’ survival**
♂ Survival curve A	708 ±34	659	100%	85%	60%
♂ Survival curve B	812 ±34	919	100%	95%	83%
**♂ Mean A&B**	**760 ±52**	789 ±130*	**100%**	90 ±5%*	71 ±11%*
♀ Survival curve A	754 ±36	713	100%	92%	64%
♀ Survival curve B	851 ±38	889	100%	95%	83%
**♀ Mean A&B**	**803 ±48**	801 ±88*	**100%**	93 ±2%*	74 ±10%*

In Survival Curve A, euthanized animals were counted as if they had died from natural causes at the time of euthanization, and in Survival Curve B, euthanized animals were considered healthy at that timepoint. This gives the interval of natural lifespan “Mean A&B”.

*Values are mean ± SEM for Survival Curve A and Survival Curve B, and for Mean A&B variation is depicted as the span between Survival Curve A and B.

**Extrapolated from curve fit

### Physiological analyses demonstrated that females have less lean mass and more body fat than males

Cohorts of mice were analyzed for behavioral phenotyping at 7, 15, and 22 months of age representing mature adults, middle-aged, and old. The mice were naïve before behavioral testing at all timepoints. As shown in Table 2, no survival loss had yet occurred at the 7-month timepoint and the mice were considered healthy adults. At the 15-month timepoint, survival was around 90% and for the final behavioral analyses at 22 months, 71% of the male and 74% of the female mice remained.

**Figure 2:**
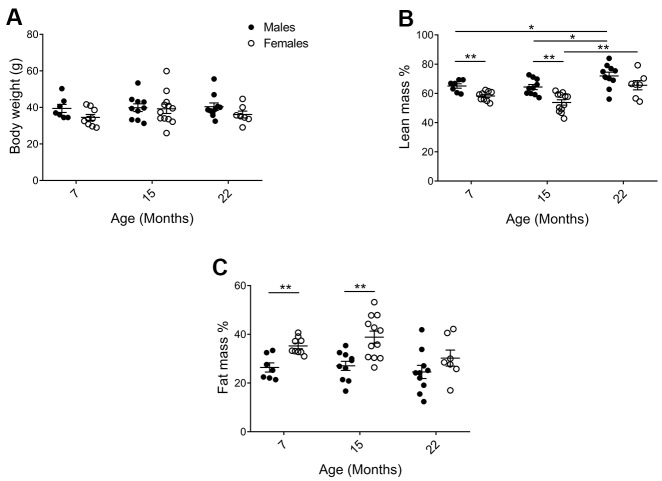
**Physiological parameters of mice in the cohorts for behavioral assessment.** (**A**) There was no difference in body weight between the cohorts, but (**B**) DEXA analysis showed that (p_M7-F7_=0.0078, p_M15-F15_=0.0011; and lean mass increased with age in males p_M7-22_=0.040, p_M15-22_=0.036; Mann Whitney), whereas (**C**) females exhibited increased fat mass as compared to males (p_M7-F7_=0.0023, p_M15-F15_=0.0012; Welch’s t-test). Values are mean ± SEM; n_M_=7, n_M15_=10, n_M22_=10, n_F7_=9, n_F15_=12, n_F22_=7.

Physiological parameters of the mice in the cohorts were assessed to validate for general health status and to identify factors that may influence behavioral analyses. As shown in Figure 2A, average body weight did not differ significantly between 7, 15, and 22 months of age or between sexes. Despite similar body weights, there were clear changes in body composition. DEXA analyses demonstrated that males had higher percent lean mass (Figure 2B; p_7_=0.0078, p_15_=0.0011) and less percent fat mass (Figure 2C; p_7_=0.0023, p_15_=0.0012), as compared to females. Percent lean mass also increased with age (Figure 2B; p_M7-22_=0.040, p_M15-22_=0.036, p_F15-22_=0.0098). At 15 months of age, the most distinct sex differences observed were an average of 11.8% more fat mass and 10.5% less lean mass in females as compared to males. Females also continuously displayed a higher core body temperature, as compared to males (Supplementary Table 2; p_7_=<0.0001, p_15_=0.0044, p_22_=0.00013). Neither bone mineral content (BMC) nor density (BMD) declined for either sex at the last timepoint (Supplementary Table 2).

### Exploratory behavior, but not necessarily activity, decreased with age

**Figure 3:**
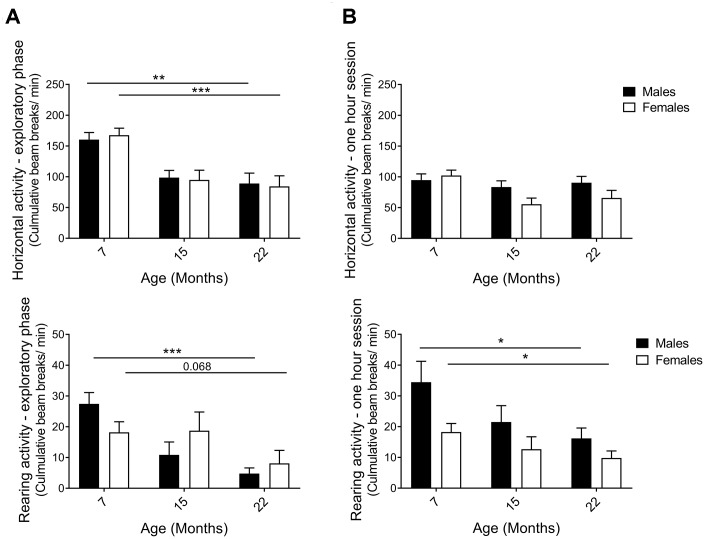
**Horizontal and rearing activity per minute in an activity box open field test.** (**A**) Activity in the first 5 minutes – the exploratory phase – decreased with aging for both males and females. The decline in horizontal (p_M7-22_=0.0065, p_F7-22_=0.0006; Student’s t-test) and rearing activities (p_M7-22_=0.0003, p_F7-22_=0.068; Mann-Whitney) indicated that exploratory behavior was highly affected by age. (**B**) Activity in 1-hour sessions of the same test. Horizontal activity was not altered from 7 to 22 months of age, whilst rearing activity declined for both female and male hybrids, thus illustrating an aging effect (p_F7-22_=0.043, p_M7-22_=0.043; Mann-Whitney). The decrease in horizontal activity observed in 15-month-old females was likely caused by different handling of this particular group due to technical reasons (see Methods). Values are mean ± SEM; n_M7_=7, n_M15_=10, n_M22_=10, n_F7_=10, n_F15_=12, n_F22_=7.

The activity box, an open-field test, was used to analyze horizontal and rearing activities of naïve hybrid female and male mice. A mouse standing on its hind limbs, termed rearing, is considered both exploratory and general vertical locomotor activity [46]. Data were separated into the first 5 minutes, representing the initial exploratory phase of the test, and the full one-hour to enable analysis of general activity and behavior in a more acquainted environment, also known as intrasessional (within session) activity. An age-effect was detected during the exploratory phase of the open-field session, with horizontal and rearing activity clearly declining from 7 to 22 months in both sexes (Figure 3A; horizontal p_M7-22_=0.0065, p_F7-22_=0.0006; rearing p_M7-22_=0.0003, p_F7-22_=0.0020). The one-hour test results demonstrated that horizontal activity did not change comparing the 7-month and the 22-month age groups of both males and females (Figure 3B; the activity was maintained through 15 months of age in males, while females exhibit an activity drop that may be caused by different handling of this particular group due to technical reasons, see Methods). Moreover, horizontal activity was found to decline within session as time elapsed in the open-field and the novelty of the environment decreased (Supplementary Figure 1A). Rearing activity over the full hour also declined with age in both males and females (Figure 3B; p_F7-22_=0.043, p_M7-22_=0.043). In contrast to horizontal activity, no differences in rearing within session were seen, *i.e*. no intrasessional changes (Supplementary Figure 1B). Thus, activity in the explorative phase was more significant and uniform in exhibiting an age-related decline than the activity during the longer sessions. Taken together, diverse exploratory behaviors decreased consistently with age in both sexes.

### Hybrid mice exhibited unaltered learning and memory with aging in the shuttle box passive avoidance test

**Figure 4:**
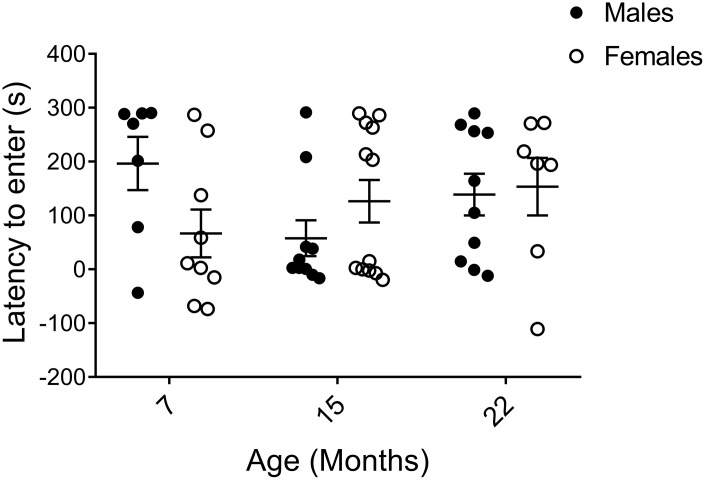
**Learning and memory assessed by shuttle box passive avoidance test.** Data are presented as the time it took to enter the conditioned area on the testing day (Day 2) subtracted by the time to enter on the conditioning day (Day 1), calculated for each individual animal, and indicate no major age or sex difference (p_M7-F7_=0.055, p_M15-F15_=0.97, p_M22-F22_=0.74, p_M7-15_=0.11, p_M7-22_=0.23, p_M15-22_=0.19, p_F7-15_=0.62, p_F7-22_=0.30, p_F15-22_=0.60; Mann-Whitney). Values are mean ± SEM; n_M7_=7, n_M15_=10, n_M22_=10, n_F7_=9, n_F15_=12, n_F22_=7.

The shuttle box passive avoidance test is a two-day procedure to assay hippocampal and amygdala-dependent learning and memory through pain conditioning [25, 30, 47, 48]. On training day, mice were allowed to enter a dark compartment, which subsequently resulted in a small electric shock. On the following day, the difference in time to re-enter the dark compartment compared to the previous day was recorded as a measure of recollection of the unpleasant experience. In order to clearly illustrate the memory response, results are presented as time to enter on Day 2 subtracted by time to enter on Day 1. Although 7-month-old males tended to keep away from the avoidance-trained area the longest, indicating the best learning and memory of all groups, large individual variations were observed within the cohorts and no statistical significances were found. Thus, we unexpectedly found no trends of declining learning and memory with age in neither sex (Figure 4).

### Increased immobility of females in forced swim test which is partially due to higher fat mass

**Figure 5:**
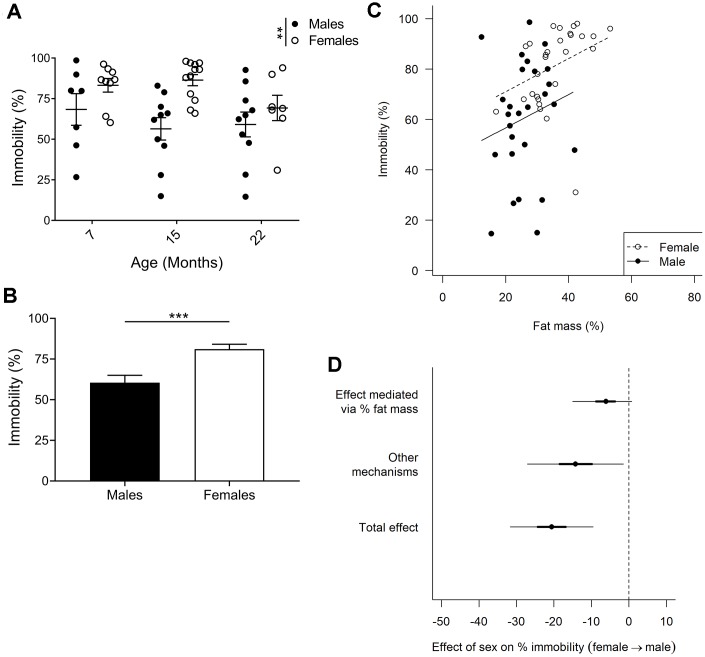
**Forced swim test to assay depressive-like behavior.** (**A**) Immobility in the forced swim test is considered an indicator of depressive-like behavior, and F2 hybrid females were found to be more immobile (p=0.0013, 2-way ANOVA), as compared to males. Values are mean ± SEM. (**B**) Due to no age effect found, all timepoints for each sex were pooled to demonstrate a 20% difference in immobility between females and males (p=0.0002, Mann-Whitney). Values are mean ± SEM. (**C**) Immobility plotted against fat mass for each animal. Females exhibited increased fat mass as compared to males, and immobility was found to correlate to percent fat mass. The correlation slopes were constrained to be equal since they were not significantly different (p=0.58, ANCOVA). (**D**) Bayesian mediation analysis separated the total effect of the difference between males and females in the forced swim test into effect mediated by fat mass or by unknown mechanisms. The sex difference in fat mass accounts for 30% of the sex difference in immobility (Bayesian p=0.96) and other mechanisms account for the remaining 70% (Bayesian p=0.99; Bayesian mediation analysis). Values are mean ±50% (thick lines) and 95% confidence interval (thin lines); n_M7_=7, n_M15_=10, n_M22_=10, n_F7_=9, n_F15_=12, n_F22_=7.

Immobility in the forced swim test is an indicator of depressive-like behavior. We did not find any age-related differences in forced swim test performance in males or females (Figure 5A; p=0.244; 2-way ANOVA)); however, when comparing males and females we observed a clear effect of sex on immobility (p=0.001; 2-way ANOVA). Since there was little evidence for a sex by age interaction (p=0.267; 2-way ANOVA), the three age groups were combined in subsequent analyses. Comparing pooled age groups demonstrated that females had around 21% increased immobility compared to males (Figure 5B; p=0.0002; Mann-Whitney).

We controlled whether body weight affected performance in all our behavioral tests but could not find any correlations. However, with females having a greater proportion of body weight comprised of fat (Figure 2C) they are likely to have increased buoyancy which may affect their performance in the forced swim test. Indeed, for both males and females, animals with a higher percent fat mass were less active (Figure 5C). We therefore used a Bayesian mediation analysis to test if, and to what extent, sex differences in the forced swim test were mediated by fat mass, and/or via other mechanisms [49, 50]. We found that fat mass accounted for about 30% of the sex effect (Figure 5D; Bayesian p=0.96; a Bayesian p-value close to 1.0 indicates that the conclusion is likely), while the remaining 70% was caused by other mechanisms (Figure 5D; Bayesian p=0.99). The differences in fat mass and immobility between males and females were most distinct at 7 and 15 months of age, despite no significant age, or age to sex interaction effect. As forced swim tests are generally not performed on aged animals, we performed the same Bayesian mediation analysis on the data set excluding the 22-month-old cohort, and found that 46% of the sex effect is caused by fat mass in this data set (Supplementary Figure 2; Bayesian p=0.986), while the difference in immobility between males and females was in the same range (24% compared to 21%). Hence, we can conclude that fat mass accounted for 30%-46% of the sex effect in the forced swim test, depending on the age groups examined.

## DISCUSSION

In this study, F2 hybrid female and male mice were assessed for behavioral tests with the aim to investigate sex differences and age-related alterations. Increasing our understanding of age-related changes in mouse behavior is vital for successful research on healthy aging. We herein: 1) reveal decreased exploratory behavior, but not altered learning/memory or depressive-like behavior, as a robust behavioral marker of aging in both male and female hybrid mice, 2) did not find sex differences in learning and memory assessed with a passive avoidance test, 3) couple immobility in a water test to body composition and demonstrate that fat mass accounted for 30-46% of the observed increase in depressive-like behavior of females compared to males, and 4) present a novel method to estimate the natural lifespan from survival studies in which animals in pain or with severe disease are euthanized.

Traditionally, lifespan studies are conducted until all animals die from natural causes and euthanization is only considered when end-stage diseases make survival for more than an additional week highly unlikely. Since aging is associated with many severe diseases, these types of experiments are especially challenging to combine with ethical considerations of animal welfare. Still, to be able to compare studies on aging phenotypes, it is crucial to distinguish the lifespan timepoint of the cohort analyzed. Here, we present a novel method that gives a qualitative estimation of the natural lifespan without the expense of animal suffering.

In our lifespan study, mice were euthanized upon signs of pain or severe disease. Counting these animals as diseased by natural cause generates an underestimation of natural lifespan, generally defined as lifetime until animals die from intrinsic causes. When euthanized animals were instead "censored" in the data analysis (i.e. counted as healthy when terminated instead of diseased), natural lifespan is overestimated since the removed animals were suffering from severe pathologies that likely limit their life expectancy. Thus, the natural lifespan is somewhere in between these lifespan calculations. Using this estimation, we categorized the 7-month-old mice as mature healthy adults with 100% survival, the 15-month-old timepoint as middle-aged with approximately 90% survival, and the 22-month-old mice as old with around 71% survival for males and 74% survival for females.

The interval of median lifespan of our C57BL/6N×BALB/c F2 male mice was in the same range as males of the four-way cross UM-HET3 mice, progeny of BALB/cByJ×C57BL/6J F1 females and C3H/HeJ×DBA/2J F1 males (789±130 compared to 742-826 days) and just below that for females (801±88 compared to 832-891 days) [51]. Compared to inbred mice, the median lifespan of both male and female C57BL/6N×BALB/c F2 mice fall in between that of the short-lived BALB/c and the long-lived C57BL/6J in the Aging Phenome Project (711/901 days for males and 771/866 days for females) [52], in which the Aging Center at the Jackson Laboratory examined the lifespan of 32 inbred mouse strains. Based on the same updated dataset [53], onset of death and maximum lifespan of our F2 males were similar to that of BALB/c males, while our F2 females more closely resembled C57BL/6J females, with similar onset of death. Additionally, only one C57BL/6J female outlived the longest-lived F2 hybrid female (the 3 longest-lived C57BL/6J females out of the 29 natural deaths died at 1180/1049/1049 days, compared to the 3 longest-lived hybrid mice out of the 13 natural deaths that died at 1051/1001/966 days).

In this study, we demonstrate that exploratory activity decreased as hybrid mice aged from 7 to 22 months. Although an age-related decline in exploratory behavior has been reported in several inbred strains, our findings confirm the pervasiveness of an age-related gradual decrease in exploratory movement for both females and males in heterogeneous hybrid mice. We thus herein have identified a decrease in exploratory behavior as a conserved non-biased behavioral hallmark of aging. Raising awareness of explorative activity in mice is vastly important in behavioral studies since the drive to explore affects fundamental incentives and can alter behavior in other tests that rely on exploration [23, 24].

In our F2 hybrid study, the mice displayed a relatively stable body weight in all age groups; likely because the first timepoint truly represented “mature adults”, and the last “old” age group represented a healthily aged cohort, thanks to the strict animal ethics protocol. Although the average body weight was comparable, female mice had more fat mass and less lean mass as compared to males, at least up to 15 months of age. In line with our results, similar body weights with increased fat mass and reduced lean mass in females as compared to males were previously observed in 20-month-old F1 hybrid C57BL/6J×129S1/SvImJ mice [54]. Additionally, body composition comparisons at 4 months of age of inbred mice demonstrated that males tended to have more lean mass, though also higher body weight, in more than half of the 40 different strains analyzed [55].

Results from this study pinpoint the importance to examine body composition in water-based behavioral studies. Fat mass content correlated to immobility in the forced swim test, which measures depressive-like behavior, with the elevated fat mass in females versus males directly accounting for 30–46% of the observed increase in depressive-like behavior. Notably, buoyancy caused by trapped air in the fur has previously been linked to swimming immobility [56], although the dependence of body fat mass in the forced swim test performance has not been identified before. We hypothesize that the increased floating sensation applied by decreased body density impacts the incentive to move. If so, fat mass likely impacts mouse activity, capacity, and behavior in water. Generally, only body weight is reported in behavioral analyses, and conclusions of depressive-like behavior using the forced swim test in obesity models, different strains, and sex comparisons have been drawn without taking body composition into account. In our study, fat mass directly accounted for 30–46% of the sex difference observed in the forced swim test, leaving 54–70% to be explained by unknown mechanisms. These may, in turn, be indirectly linked to fat mass through physiological effects. One example of this could be a reduced contractile function of muscle reported in obese mice [reviewed in 57], a finding that may also restrict water mobility in females. There are a number of potential fat mass unrelated mechanisms that could also play a role, including sex hormone levels as well as hormone signaling and their effects on neuronal circuits (though both of these examples are known to interact with metabolism). Taken together, we can only speculate and cannot exclude that females exhibited enhanced depressive-like behavior regardless of body composition. However, the 30-46% effect due to fat mass could still be the single most important factor on increased depressive-like behavior in females as compared to males. In addition, we did not identify any mobility alterations upon aging in the forced swim test in either males or females.

We found no significant sex or age effect on memory of passive avoidance learning using a one-time pain-conditioning factor. To our knowledge, only Benice and colleagues [30] have investigated aging in female and male mice using a passive avoidance test prior to us. In that study, inbred mice were conditioned the first day until they did not enter the dark compartment for three consecutive trials with a maximum of 10 trials [30]. Although it was stated that there were no significant age or sex differences regarding the conditioning trials, the cognitive learning and memory responses after multiple electric shocks enforces amygdala over hippocampal dependency of the test and increases memory consolidation [58, 59], and thus convolutes possible comparisons and extrapolations to our study. Nonetheless, our data are in line with active avoidance and object memory consolidation tests of learning and memory in inbred mice, in which females and males performed equally well [10, 23, 30, 35]. We hypothesize that different age-related cognitive impairments are reflected depending on the test used. In order to resolve any controversies surrounding sex differences in various learning and memory tests, further analyses are needed to ensure that spatial testing is not sex-biased for reference cues. Moreover, we also strongly recommend that body composition and buoyancy are taken into account when analyzing results from water-based cognition tests, including the commonly used Morris water maze.

In summary, this work is the first behavioral phenotypic aging study to use hybrid mice and include analyses of both sexes. We herein have confirmed that decreased exploratory behavior is a conserved robust marker of aging, while no observable differences in general activity or in memory of passive avoidance learning were found. We have also demonstrated that increased fat mass partly explains why females swim less than males in the forced swim test of depressive-like behavior. This novel finding emphasizes the need to control for body composition in water-based tests. Moreover, we present a new method to qualitatively estimate natural lifespan in which animals are euthanized upon pain or severe disease.

## METHODS

### Animals and diets

Subjects were male and female C57BL/6N×BALB/c F2 hybrid mice crossed from C57BL/6N×BALB/c F1 hybrids, with C57BL/6N fathers (Charles River, Lyon, France) and BALB/c mothers (Harlan Laboratories, Horst, the Netherlands), with coat color ranging from white to brown and black. Males were cohoused in groups of three until 6 months of age. In 11 cages with male mice, fighting led to severe injury and all animals in those cages were removed from the study. This was done in order to maintain a representative population, since high-ranked animals would otherwise be selected for. All males were single-housed for the remaining time from 6 months of age. Females were cohoused in groups of four, with the number of animals per cage decreasing as mice deceased in the lifespan study. The mice were not pooled. Cages were equipped with nesting material (paper), cardboard houses, and wooden sticks, and were cleaned every two weeks for single housed males and every week for cohoused mice. Old nesting material was transferred to the new cages upon cleaning. The mice had *ad libitum* access to regular chow diet (R3; Lactamin, Kimstad Sweden) containing 12% fat, 62% carbohydrates, and 26% protein (energy percentage), with a total energy content of 3 Kcal/g. The mice were maintained on a 12:12 h light/dark cycle (gradual light increase from 5:30-6:00 am and decrease at 5:30–6:00 pm) at 21°C. All experimental protocols of the study were carried out in accordance with the ethical certificate approved by the Animal Ethics Committee in Gothenburg, Sweden (Permit Number: 164-2015) and with EU Directive 2010/63/EU for animal experiments. Causes of euthanization were symptoms indicative of severe ill-health. These include: hunched shoulders, shabby fur, inactivity, failure to eat or drink, enlarged organs and tumors. Upon termination, mice were euthanized by decapitation under 5% isoflurane anesthesia.

### Study design of physiological and behavioral phenotypic profiling

The lifespan study consisted of 20 male and 22 female mice, which were not part of the behavioral testing cohorts. Littermates to these mice were tested for physiological and behavioral phenotypic profiling at three ages representing mature adults (7 months; 7 males and 10 females), middle-aged (15 months; 10 males and 12 females) and aged (22 months; 10 males and 7 females) [60]. Specifically, the mice’s ages at behavioral profiling were 6.6-7.8±0.2 (age at test period start - age at test period end ±SD of age variance in the cohort; 7 months), 14.5-15.6±0.1 (15 months), 21.8-22.5±0.2 (22 months). These mice were initially included in the lifespan analysis but taken out (marked as censored) at the testing timepoint, and thus were only included in the calculation of percent survival in the lifespan analysis until their removal. Results from the 7-month cohorts served as control in a previous study [61]. Test periods occurred at different times of the year without any attempt to control for seasonal effects (7-month-olds: September-October; 15-month-olds: May-June; 22-month-olds: late December - January). Prior to the testing period, all mice were handled and acclimated for one week. The sequence of analyses is provided in Supplementary Table 3. Due to technical and practical reasons in our animal facility, the order of the behavioral tests could not be identical for males and females for all timepoints. Behavioral tests that are known to be impacted by handling (such as activity box) were performed early in the testing period (except for the 15-month-old female activity box timepoint; conclusions from which are therefore omitted). No correlation between behavioral test results and coat color could be found. Tests that affect the mice to a greater extent were planned later in the testing periods and/or the animals were given an extended recovery time in order to not impact the subsequent analyses. All animals were acclimated to the behavioral testing room for at least one hour prior to testing, and all experiments started between 10-11 am with the time spent for each experiment noted for each individual test. One female in the 7-month timepoint was removed from testing due to an eye injury.

### Body composition and core temperature measurements

Core body temperature, body weight, and body composition were obtained at the same time. Core body temperature was taken with a rectal probe thermometer (ELFA AB, Sweden) followed by dual energy X-ray absorptiometry (DEXA) scanning (Lunar PIXImus Densitometer, GE Medical Systems, Madison, WI, USA) while under 2.5% isoflurane sedation for approximately 4 minutes. The parameters recorded were: body length (cm), body fat (g), lean body mass (g), bone mineral density (BMD; g/cm2), and bone mineral content (BMC; g) [47].

### Activity box

The activity box is an open-field activity-like test to assess general activity, exploratory behavior, and signs of anxiety [47]. The activity box records movement of each mouse three dimensionally using infrared sensors built into the walls (8Lx8Bx8H) of a sound-proof opaque box (50x50x50 cm) with a low intensity lamp over the lid (Kungsbacka mät och regler, Fjärrås, Sweden). The mice were placed in the middle of the box and their movements were recorded for a total of 1 hour. The first 5 minutes were used to assess exploratory behavior in this novel environment and the full hour was used to assess general activity and behavior. The parameters that were recorded as events every 5 minutes include: horizontal activity, peripheral activity, rearing activity, peripheral rearing, rearing time, locomotion, and corner time.

### Forced swim test

The forced swim test is used to analyze mice for signs of depression [47, 62, 63]. A transparent plexiglas cylinder (25 cm inner-diameter, 60 cm length) was filled with room temperature (22 °C) water level with a grey circular plastic platform hanging from wires on the outside of the cylinder, approximately 20 cm from the top (bespoke construction, AstraZeneca, Gothenburg, Sweden). A single mouse was placed on the water surface and monitored for 6 minutes and 20 seconds by a video camera placed directly above the cylinder, with the last 4 minutes used to analyze behavior and activity (MouseTracker analysis software, Mölndal, Sweden).

### Shuttle box passive avoidance test

The shuttle box passive avoidance test is used to study memory performance in mice [47, 64]. The shuttle box system (Accuscan Instruments Inc., Columbus, OH, USA) is made up of a cage centrally divided by a wall with a sliding door creating two compartments, one bright with transparent walls and one dark covered on all sides with opaque walls. Both compartments are equipped with sensors that determine the location of the mouse, and the floor of the cage is made of a stainless-steel grid that can deliver a mild electric shock. On the first day, each mouse was placed into the bright compartment, and after 60 seconds the central door opened allowing the mouse to migrate into the dark compartment, an environment which they should prefer. Upon entry into the dark compartment, immediately after the door closed, the mouse received a mild electric shock (0.3 mA) and remained in the dark compartment for at least 30 seconds thereafter. On the second day, the mouse was again placed into the bright compartment and after 60 seconds when the central door opened the mouse was given the same choice to enter into the dark compartment (300 seconds maximum time). The time of entry into the dark compartment was recorded for both days, and no entry or entry after a longer interval on the second day as compared to the first day was considered a memory response.

### Statistical analysis

Comparisons between two groups were analyzed for normal distribution by Shapiro-Wilks and Levene’s test for homogeneity of variance. Two-tailed independent t-test (Student’s t-test) was used for groups that met these criteria, two-tailed independent unequal variance t-test (Welch’s test) for normal distributed groups which failed Levene’s test, and nonparametric Mann-Whitney U test for unevenly distributed groups. Comparisons of survival curves were analyzed with the log-rank Mantel-Cox test. Interactions and comparisons of age and sex effects were calculated with 2-way ANOVA. Covariance was analyzed with ANCOVA. Differences were considered significant at *P* < 0.05. A Bayesian mediation analysis was used to test whether sex differences on the forced swim test were due to difference in fat mass between sexes, or due to other mechanisms [described in 49, 50]. The Bayesian p-value reported in the results has a direct and intuitive interpretation: it is the probability of the conclusion given the data, so a high value means that the conclusion is highly probable. Statistics were calculated using IBM SPSS Statistics 25, GraphPad Prism 7, or R/Stan.

## Supplementary Material

Supplementary Figures

Supplementary Tables
