# Supplementary material for: Conclusions from a behavioral aging study on male and female F2 hybrid mice on age-related behavior, buoyancy in water-based tests, and an ethical method to assess lifespan
Source: Aging (Albany NY). 2019 Sep 11;11(17):7150–68. doi: 10.18632/aging.102242 (PMC6756906; doi:10.18632/aging.102242)
Supplement: Supplementary Figures [file aging-11-102242-s002.pdf]

SUPPLEMENTARY FIGURES

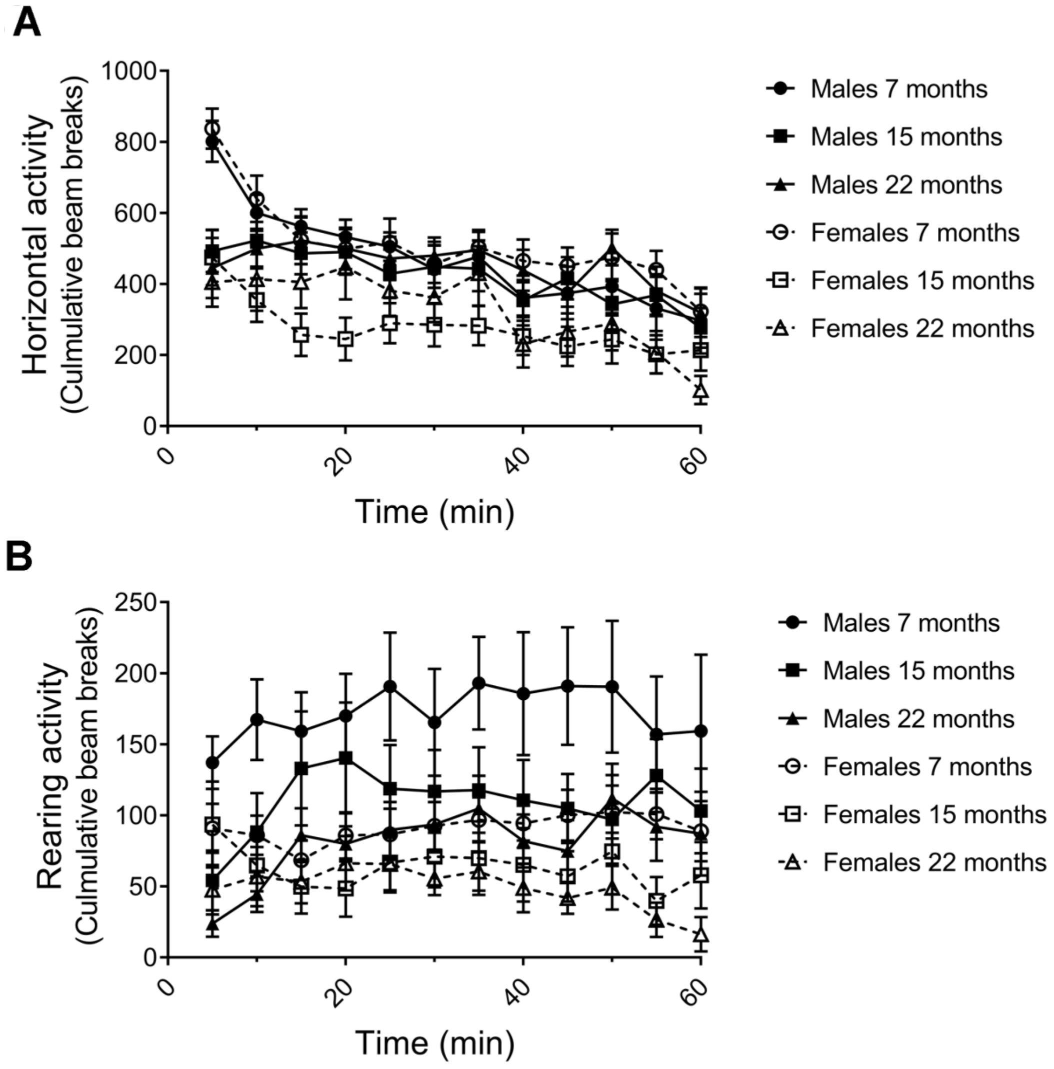

Supplementary Figure 1. One-hour open field test activity in 5-minute bins. (**A**) Horizontal activity declined intrasessionally for all cohorts, 7-month-old males and females demonstrated augmented activity in the initial phase of testing. (**B**) Rearing activity decreased with age but is maintained throughout the test hour regardless of age. Data represents mean ± SEM. n_{M7}=7, n_{M15}=10, n_{M23}=10, n_{F7}=10, n_{F15}=12, n_{F23}=7

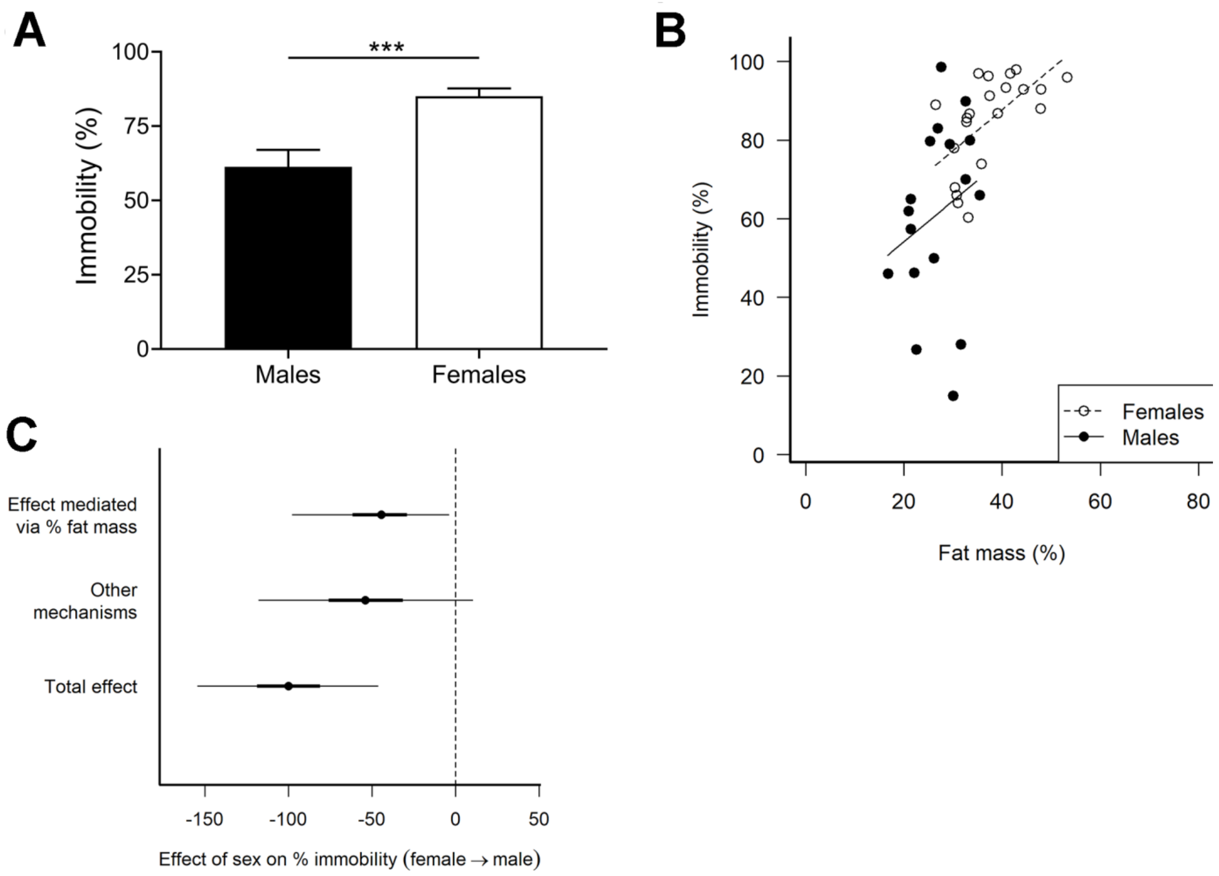

Supplementary Figure 2. Immobility in the forced swim test for 7- and 15-month-old males and females. (**A**) The sex difference in immobility when pooling females and males from the 7- and 15-month-old timepoints was 24% as compared to 21% when including 23 months animals (p=0.0007, Mann Whitney). Data represents mean ± SEM. (**B**) Immobility plotted against fat mass for each animal from the 7 and 15 months' timepoints confirms the correlation of fat mass to immobility. (**C**) Since the sex difference in immobility remained similar (21–24%) but fat mass differences were most pronounced at 7 and 15 months, Bayesian mediation analysis was recalculated to answer how much of the effect was mediated by fat mass at 7 and 15 months. Results demonstrated that the effect mediated by fat mass increased to 46% (p=0.986) of the total immobility effect and other unknown mechanisms accounted for 54% (p=0.952) when older animals were excluded. This signifies that even though the sex difference is most likely independent of age, the effect mediated by fat mass is greater at younger ages (46% compared to 30%). Values are mean ±50% (thick lines) and 95% confidence interval (thin lines); n_{M7}=7, n_{M15}=10, n_{F7}=10, n_{F15}=12.
