# Supplementary material for: Conclusions from a behavioral aging study on male and female F2 hybrid mice on age-related behavior, buoyancy in water-based tests, and an ethical method to assess lifespan
Source: Aging (Albany NY). 2019 Sep 11;11(17):7150–68. doi: 10.18632/aging.102242 (PMC6756906; doi:10.18632/aging.102242)
Supplement: Supplementary Tables [file aging-11-102242-s001.pdf]

SUPPLEMENTARY TABLES

Supplementary Table 1. Order of deaths in lifespan study.

Age (days)	Animal	Cause of death	Age (days)	Animal	Cause of death
183	599, 2 (M), Brown	Timepoint 7 months	444	508, 5 (M), Brown	Timepoint 15 months
207	387, 1 (M), Black,	Timepoint 7 months	444	153, 1 (F), Brown	Timepoint 15 months
207	388, 1 (M), Brown	Timepoint 7 months	444	154, 2 (F), Brown	Timepoint 15 months
207	392, 5 (M), Brown	Timepoint 7 months	444	156, 2 (F), Albino	Timepoint 15 months
207	226, 2 (F), Brown	Timepoint 7 months	444	157, 3 (F), Albino	Timepoint 15 months
207	228, 4 (F), Brown	Timepoint 7 months	444	163, 3 (F), Brown	Timepoint 15 months
207	230, 1 (F), Albino	Timepoint 7 months	444	165, 5 (F), Brown	Timepoint 15 months
207	231, 2 (F), Albino	Timepoint 7 months	444	271, 2 (F), Brown	Timepoint 15 months
207	341, 1 (F), Brown	Timepoint 7 months	444	273, 4 (F), Brown	Timepoint 15 months
207	342, 2 (F), Brown	Timepoint 7 months	444	274, 5 (F), Brown	Timepoint 15 months
207	406, 6 (F), Brown	Timepoint 7 months	444	281, 4 (F), Albino	Timepoint 15 months
207	384, 7 (F), Brown	Timepoint 7 months	445	169, 3 (M), Brown	Timepoint 15 months
207	459, 10 (F), Brown	Timepoint 7 months	445	174, 3 (M), Albino	Timepoint 15 months
208	184, 1 (M), Brown	Timepoint 7 months	445	175, 1 (F), Black	Timepoint 15 months
208	185, 2 (M), Brown	Timepoint 7 months	445	176, 2 (F), Black	Timepoint 15 months
208	187, 4 (M), Brown	Timepoint 7 months	499	411, 3 (M), Brown	Removed: skinny, wound
208	419, 5 (F), Albino	Timepoint 7 months	560	420, 1 (F), Black	Natural death
308	507, 4 (M), Brown	Removed: testicular tumor	570	243, 3 (F), Brown	Removed: skinny
311	530, 5 (M), Brown	Natural death	570	220, 4 (M), Brown	Removed: abdominal tumor
312	532, 2 (M), Albino	Natural death	584	365, 2 (F), Albino	Removed: ovarian tumor
322	171, 5 (M), Brown	Removed: skinny, dehydrated	608	471, 8 (M), Brown	Removed: large spleen
352	192, 2 (F), Brown	Natural death	613	610, 3 (M), Brown	Natural death
401	288, 4 (F), Brown	Removed: enlarged spleen	620	539, 3 (M), Brown	Natural death
406	321, 3 (F), Brown	Natural death	626	376, 1 (F), Black	Natural death
442	246, 1 (M), Brown	Removed: enlarged spleen	626	383, 6 (F), Brown	Removed: tumor eye
431	564, 1 (M), Brown	Timepoint 15 months	626	462, 3 (F), Albino	Removed: skinny
435	524, 1 (M), Black	Timepoint 15 months	626	474, 3 (M), Albino	Removed: testicular tumor
435	525, 2 (M), Black	Timepoint 15 months	627	216, 2 (F), Albino	Removed: very skinny
444	527, 2 (M), Brown	Timepoint 15 months	627	252, 1 (F), Brown	Natural death
444	347, 1 (M), Brown	Timepoint 15 months	637	247, 2 (M), Brown	Removed: skinny
444	349, 3 (M), Brown	Timepoint 15 months	646	540, 4 (M), Brown	Removed: large spleen
444	505, 2 (M), Brown	Timepoint 15 months	647	538, 2 (M), Brown	Natural death

653	573, 2 (M), Brown	Timepoint 23 months	667	213, 1 (F), Brown	Timepoint 23 months
656	537, 1 (M), Brown	Timepoint 23 months	667	200, 2 (F), Brown	Removed: impaired activity
656	542, 6 (M), Brown	Timepoint 23 months	667	201, 3 (F), Brown	Removed: enlarged abdomen, large spleen
656	543, 7 (M), Brown	Timepoint 23 months	671	622, 4 (M), Brown	Timepoint 23 months
658	473, 2 (M), Albino	Removed	713	191, 1 (F), Brown	Removed: ovarian tumor
659	409, 1 (M), Brown	Removed: large spleen	751	181, 5 (F), Brown	Natural death
666	257, 2 (M), Brown	Timepoint 23 months	819	566, 3 (M), Brown	Natural death
666	259, 4 (M), Brown	Timepoint 23 months	860	591, 4 (M), Brown	Removed
666	261, 6 (M), Brown	Timepoint 23 months	888	319, 1 (F), Brown	Natural death
666	232, 3 (F), Albino	Timepoint 23 months	889	287, 3 (F), Brown	Natural death
666	239, 1 (F), Black	Timepoint 23 months	911	368, 1 (M), Brown	Removed: skinny, impaired activity
666	514, 2 (F), Black,	Timepoint 23 months	919	608, 1 (M), Brown	Natural death
666	522, 1 (F), Albino	Timepoint 23 months	923	385, 1 (F), Albino	Natural death
667	195, 1 (M), Brown	Timepoint 23 months	936	523, 2 (F), Albino	Natural death
667	248, 3 (M), Brown	Timepoint 23 months	966	285, 1 (F), Brown	Natural death
667	407, 1 (M), Black	Timepoint 23 months	1001	179, 3 (F), Brown	Natural death
667	200, 2 (F), Brown	Timepoint 23 months	1051	499, 1 (F), Brown	Natural death
667	201, 3 (F), Brown	Timepoint 23 months			

Individual data of each mice in the lifespan analysis; age (days), animal number, sex, coat color and indication of death. The ages of the mice were exactly 6.6-7.8±0.2, 14.5-15.6±0.1 and 21.8-22.5±0.2 months±SD. Lifespan analysis: n_{males}=48, $n_{females}$=51, including lifespan study: n_{males}=20, $n_{females}$=22 and behavioral timepoints: n_{M7}=7, n_{M15}=10, n_{M22}=10, n_{F7}=9, n_{F15}=12, n_{F22}=7.

Supplementary Table 2. Additional physiological parameters.

		BL	Body Temp	BMD	BMC	Fat mass	Lean mass
		(cm)	(ºC)	(mg/cm^2)	(g)	(g)	(g)
MALES	7	10.8 ± 0.11[2]	36.7 ± 0.18[1,2]	56.5 ± 1.34	0.62 ± 0.03	9.4 ± 1.2[2]	25.5 ± 0.83[1,2]
	15	10.7 ± 0.11	37.2 ± 0.11[1,2]	57.1 ± 1.05	0.65 ± 0.02[2]	9.9 ± 1.2[2]	25.4 ± 0.98[1,2]
	22	10.8 ± 0.11	36.5 ± 0.13[1,2]	56.3 ± 1.13	0.65 ± 0.02	9.9 ± 1.7	28.7 ± 0.46[1,2]
FEMALES	7	10.3 ± 0.09[2]	38.4 ± 0.16[1,2]	56.8 ± 0.84	0.56 ± 0.02	11.0 ± 0.8[2]	20.0 ± 0.59[1,2]
	15	10.5 ± 0.11	37.6 ± 0.10[1,2]	49.3 ± 4.58	0.51 ± 0.04[2]	14.1 ± 1.8[2]	20.7 ± 0.85[1,2]
	22	11.0 ± 0.12	37.7 ± 0.18[1,2]	56.8 ± 1.35	0.63 ± 0.04	10.6 ± 1.6	23.5 ± 1.02[1,2]

Body composition and core temperatures were analyzed by DEXA and rectal thermometer. [1)]Statistical significance within sex, [2)]statistical significance between sexes. Body length [2)]$p_{M7\text{-}F7}$=0.0040, body temperature [1)]$p_{M7\text{-}15}$=0.055, $p_{M7\text{-}15}$=0.055, $p_{F7\text{-}15}$=0.0017, $p_{M15\text{-}22}$=0.00072, $p_{F15\text{-}22}$=0.017; [2)]$p_{M7\text{-}F7}$=>0.0001, $p_{M15\text{-}F15}$=0.0044, $p_{M22\text{-}F22}$=0.00013, bone mineral density (BMD), bone mineral content (BMC) [2)]$p_{M15\text{-}F15}$=0.0060, fat mass [2)]$p_{M7\text{-}F7}$=0.0023, $p_{M15\text{-}F15}$=0.0012 (Welch's t-test), and lean mass [2)]$p_{M7\text{-}F7}$=0.0002, $p_{M15\text{-}F15}$=0.0006, $p_{M22\text{-}F22}$=0.0007, [1)]$p_{M7\text{-}22}$=0.0061, $p_{M15\text{-}22}$=0.0095, $p_{F7\text{-}22}$=0.011, $p_{F15\text{-}22}$=0.023 (Mann-Whitney). n_{M7}=7 n_{M15}=10 n_{M22}=10 n_{F7}=9 n_{F15}=12 n_{F22}=7 Mean ± SEM.

Supplementary Table 3. Order of behavioral tests at 7-, 15-, and 22-month-old timepoints.

MALES			FEMALES		
7	15	22	7	15	22
FST	OFT	DEXA	OFT	DEXA	OFT
OFT	DEXA	OFT	FST	FST	PAT
PAT	PAT	FST	PAT	PAT	DEXA
DEXA	FST	PAT	DEXA	OFT	FST

Behavioral and physiological analyses: forced swim test (FST), activity box – open field test (OFT), shuttle-box passive avoidance test (PAT), dual energy X-ray absorptiometry (DEXA). n=7-12 per group
